# Longitudinal Assessment of Antisaccades in Patients with Multiple Sclerosis

**DOI:** 10.1371/journal.pone.0030475

**Published:** 2012-02-02

**Authors:** Joanne Fielding, Trevor Kilpatrick, Lynette Millist, Meaghan Clough, Owen White

**Affiliations:** 1 School of Psychology and Psychiatry, Monash University, Clayton, Victoria, Australia; 2 Department of Neurology, Royal Melbourne Hospital, Parkville, Victoria, Australia; 3 Centre for Neuroscience, University of Melbourne, Parkville, Victoria, Australia; Institute Biomedical Research August Pi Sunyer (IDIBAPS) - Hospital Clinic of Barcelona, Spain

## Abstract

We have previously demonstrated that assessment of antisaccades (AS) provides not only measures of motor function in multiple sclerosis (MS), but measures of cognitive control processes in particular, attention and working memory. This study sought to demonstrate the potential for AS measures to sensitively reflect change in functional status in MS. Twenty-four patients with relapsing-remitting MS and 12 age-matched controls were evaluated longitudinally using an AS saccade task. Compared to control subjects, a number of saccade parameters changed significantly over a two year period for MS patients. These included saccade error rates, latencies, and accuracy measures. Further, for MS patients, correlations were retained between OM measures and scores on the PASAT, which is considered the reference task for the cognitive evaluation of MS patients. Notably, EDSS scores for these patients did not change significantly over this period. These results demonstrate that OM measures may reflect disease evolution in MS, in the absence of clinically evident changes as measured using conventional techniques. With replication, these markers could ultimately be developed into a cost-effective, non-invasive, and well tolerated assessment tool to assist in confirming progression early in the disease process, and in measuring and predicting response to therapy.

## Introduction

Multiple sclerosis is a multifocal inflammatory demyelinating disease, with a pathophysiological process that involves the interplay of damage and repair mechanisms. During acute relapse, inflammatory demyelination causes conduction block and slowing of action potentials leading to physical symptoms. Once resolved, processes such as remyelination, sodium channel reorganisation and cortical plasticity contribute to recovery of function. However, through accumulation of residual damage and degenerative changes the disease course eventually transforms into uninterrupted progression. These processes vary considerably both within and between patients, resulting in a highly unpredictable clinical course.

Unfortunately, current clinical assessments provide a crude measure of functional change in MS patients. The most commonly used clinical tool, the Kurtzke Expanded Disability Scale (EDSS) [1], is widely criticised as a blunt instrument, with scores largely biased towards pyramidal function. Not only is the EDSS relatively insensitive to change, it provides little information about other important functions, particularly cognitive function and the integration of sensory and cognitive processes. Cognitive changes are present in up to 70% of MS patients [2], [3], even where diagnosis is only probable [4]. Further, the correlation between the clinical manifestations of the disease and the burden of lesions observed on conventional magnetic resonance imaging (MRI) scans remains weak. Not only are white matter (WM) lesions often clinically silent, but conventional techniques offer a limited capacity to characterize and quantify the heterogeneous features of MS pathology, occult changes in normal appearing white matter as well as damage to grey matter. Significantly, brain volumes reduce regardless of continuing inflammatory activity, demonstrating an important neurodegenerative component.

While more modern quantitative MRI techniques have the potential to overcome some of the limitations of conventional measures, their cost, availability, complexity and lack of validation limit their use in routine clinical practice [5]. It is therefore crucial from a clinical perspective, that we concurrently develop user-friendly, cost-effective surrogate markers of disease activity that provide sensitive assessment of functional change. This is particularly important as new therapies evolve.

We have previously demonstrated that assessment of ocular motor (OM) function provides not only a measure of lower level motor control processes, but of impaired cognitive control processes in MS [6], [7], [8]. In a cross-sectional study of 25 patients, attentional and working memory deficits were reflected in difficulty inhibiting a response to a non-target stimulus, protracted and more variable movement onsets, and poor spatial accuracy. Importantly, these deficits correlated significantly with scores on neuropsychological tests commonly used in MS, including the Paced Auditory Serial Addition Task (PASAT), considered the reference task for the cognitive evaluation of MS patients [9].

This study sought to demonstrate the potential for OM measures to sensitively reflect change in functional status in MS, whether functional decline or recovery. A group of patients with relapsing-remitting MS (RRMS) and age-matched control were retested on the AS task approximately two years following initial testing (*M* = 23.83 mths, range 23–29 mths). We propose that these measures may ultimately provide para-clinical or surrogate markers of (dys)function which can be used to assess relapse, progression, recovery, and the consequences of therapeutic intervention in MS.

## Materials and Methods

### Ethics statement

Ethics approval was granted by the Melbourne Health Human Research Ethics Committee and all participants gave written informed consent prior to inclusion in the study, in accordance with the Helsinki declaration.

### Participants

Twenty four patients meeting the McDonald criteria for RRMS participated in this study. Mean age at follow-up was 41 years (range 26–56 years), mean disease duration was 87 mths (range 28–200 mths), and scores using the Expanded Disability Status Scale (EDSS) ranged from 0–5 (median score of 1). Ten patients had a clinical score of 0 on the EDSS. Eleven patients experienced new episodes in the interim between testing sessions, although none were retested within 3 months of a relapse. Twelve age-matched neurologically healthy individuals [mean age of 43 years (range 29–64 years)] served as a control group. Exclusion criteria for control subjects were a history of head injury, central neurological disorder or psychiatric illness, drug abuse, or regular intake of psychoactive drugs. All MS patients continued with their normal medication regime.

### Equipment

Horizontal displacement of the eye was recorded using an IRIS infrared eye tracker (Skalar Medical, BV, Delft, The Netherlands), with output sampled at 1 kHz. Screen based stimuli were displayed on a 21 inch monitor (Mitsubishi Electric Corporation, Tokyo, Japan), and generated using E-Prime software (Psychology Software Tools, Inc, PA, USA). Participants were seated 840 mm directly in front of the monitor with their heads stabilized using a custom-made bite bar. Test stimuli were presented on a black background and comprised green targets (cross; 30 mm×30 mm) presented centrally, 5° or 10° from centre in either hemifield, and a white centrally-positioned re-fixation stimulus (square ring, 10 mm×10 mm). Output from the eye tracker was displayed alongside a control signal generated by E-Prime, which indicated stimulus change. A photodiode was placed directly over a non-visible portion of the screen to concurrently record stimulus change in real-time.

### Antisaccade task

Participants were instructed to fixate a centrally-positioned target. Following a period of either 1250 or 1600 msec, this target was extinguished coincident with the appearance of a peripheral target. Participants were instructed not to look at the peripheral target but to make a saccade in the opposite direction as quickly and accurately as possible, ending an equal distance from the centre of the screen. The peripheral target was extinguished after 1500 msec and a re-fixation stimulus, presented for 150 msec, redirected gaze back to centre prior to the onset of the next trial. The task included 48 trials (24 left, 24 right, balanced for 5° and 10° steps).

### Neuropsychological tests

Attention, working memory, and speed of information processing were assessed using the Paced Auditory Serial Addition Task (PASAT). The PASAT is considered the reference task for the cognitive evaluation of MS patients, and involves the presentation of single digits presented every 3 (or 2) seconds with the participant adding each new digit to the one immediately prior to it. Depressive symptoms were evaluated using the Beck Depression inventory (Beck and Steer, 1991). Standardised instructions were followed

### Statistical analyses

Key measures were proportion of errors (to non-target stimuli), saccade latency, and mean absolute position error [(EP_final_−SP)/SP]×100, where Ep_final_ is the final eye position and SP is the stimulus position. Absolute change over time was calculated for all variables and means for MS patients and control subjects were compared using standard parametric (t-test or Welch test where a violation of the assumption of equal variance was evident) and non-parametric tests where appropriate (Mann-Whitney U). Neuropsychological tests scores were correlated against OM measures for MS patients using Pearson's r.

## Results

### Means

**Figure 1:**
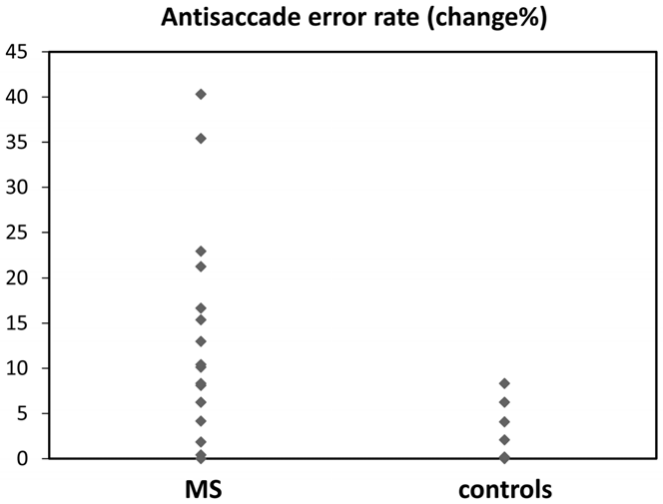
Change in proportion of AS errors over two years.

**Figure 2:**
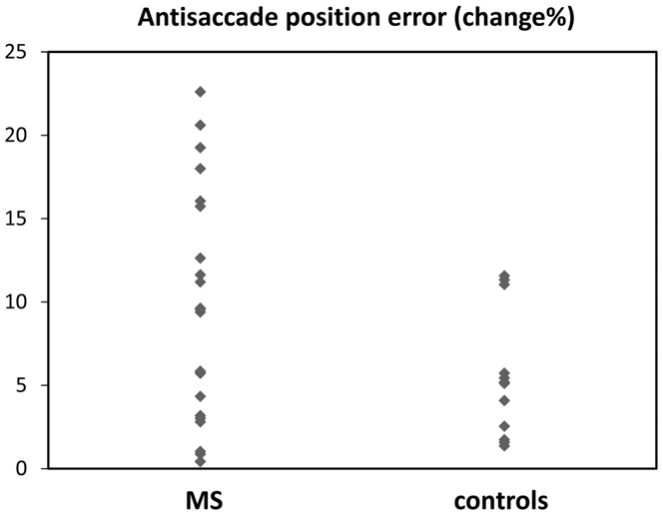
Change in AS position error over two years.

An important finding here was that for control subjects, no significant change was found over two years for any experimental variable. However, compared to control subjects, changes in AS saccade parameters were significantly greater for MS patients (see Table 1): these included AS error rate, *U* = 66.5, *z* = −2.33, *p*<0.05 (see Figure 1), and AS latency, *U* = 69, *z* = −2.12, *p*<0.05, with a near significant difference in AS position error, *t*(34) = 1.89, *p* = 0.06 (see Figure 2). Of note, EDSS scores for these patients did not change significantly over the two year period, and did not correlate with any experimental variable (mean EDSS = 1.46 at entry, and 1.14 at two years, *p* = 0.282)

**Table 1:** Mean absolute differences in saccade parameters over two years.

	MS	Controls
AS Errors (%)	12.94	6.33
AS Latency (msec)	34.40	17.90
AS Position error (%)	8.92	5.56

### Correlations

**Figure 3:**
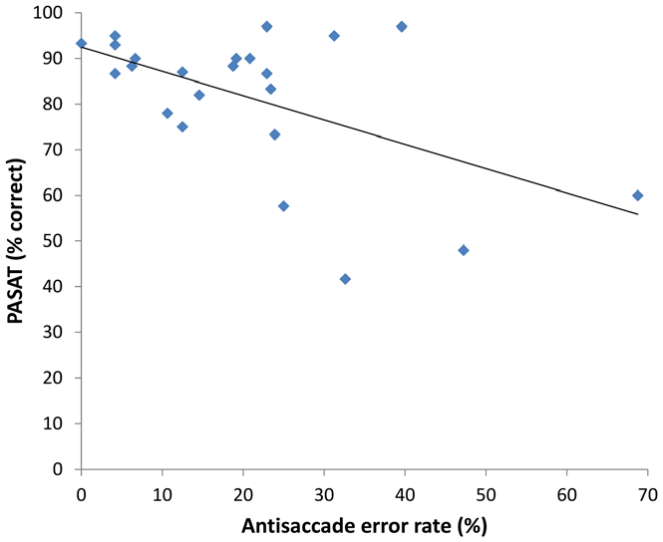
Correlation between AS errors and PASAT scores in MS patients at two years.

**Figure 4:**
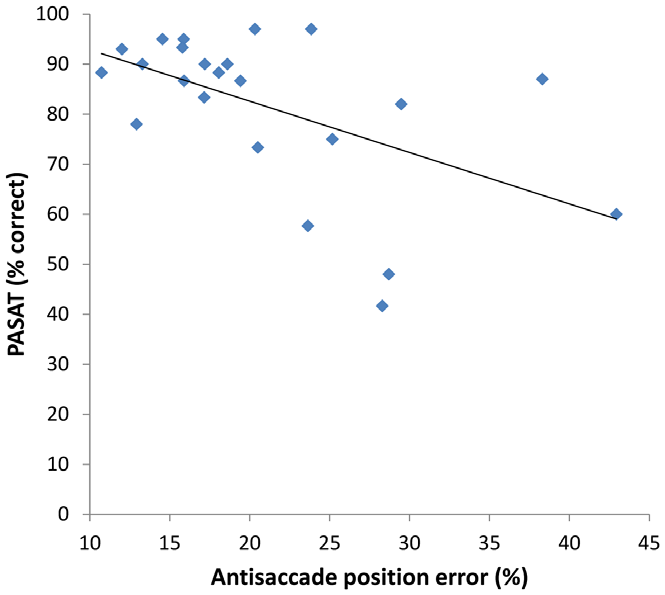
Correlation between AS position error and PASAT scores in MS patients at two years.

For MS patients, significant correlations were revealed between PASAT scores (% correct) and AS error rate (*r* = −.54, *p*<0.01), and AS position error (*r* = −.54, *p*<0.01), see Figures 3 and 4.

## Discussion

In a two-year follow-up study of RRMS patients we found that, compared to control subjects, a range of saccade parameters altered significantly, in particular those proposed to reflect attentional and working memory processes. Importantly, these alterations were evident in the absence of any clinically evident change in functional status, and associations with neuropsychological assessments were maintained over time. In particular, error and accuracy measures retained correlation with scores on the PASAT, a complex test of executive function, working memory, information processing speed and divided attention, commonly used in MS.

Although saccadic dysmetria, internuclear ophthalmoplegia, and fixation instability are clinically recognised features of MS [10], each depend largely on the integrity of defined areas of the brainstem. However, the control of more volitional saccadic eye movements, like those investigated here, involves a reciprocally connected, and widely distributed cortical/sub-cortical network, which integrates complex afferent and efferent information to generate appropriate, context-specific responses. Like cognitive function more generally, diffuse disturbance of long-range links, as would be expected in MS, disrupts activity inside this large, distributed network [11], [12], [13]. Subsequently, change, whether a function of further damage or repair, are reflected in altered performance.

As the clinical and radiological course of MS is highly variable, this group of patients necessarily comprised individuals whose functional status had stabilised, improved or deteriorated, hence our reporting absolute change. However, we would anticipate that assessment over a significantly longer period would reflect accumulated deficit, thus functional decline using our select OM measures. Interestingly, a 2-year follow-up study by Derwenskus et al (2005) [10] using observational analyses of OM function, revealed an increase in deficits not represented by Kurtzke (EDSS) scores of brainstem or cerebellar function (e.g. number of inaccurate gaze shifts, number of saccadic intrusions). Like our group, many of these patients had an otherwise full range of movement, and therefore deficits would not have been detected using conventional measures.

As for any useful biomarker in MS, we have demonstrated that OM measures are accurate, reproducible and highly sensitive to change in disease status. Notably, deficits were found irrespective of initial clinical status, even in patients who scored ‘0’ on the EDSS. We have also demonstrated correlation with other, validated disease measurements. Measurement of eye movements is cost-effective, non-invasive, and well tolerated. With replication in larger, well-defined cohorts, these markers could be developed to complement other diagnostic and exploratory studies, affording a degree of quantification not found in methods currently used in MS.
